# Resistin-Induced Endoplasmic Reticulum Stress Contributes to the Impairment of Insulin Signaling in Endothelium

**DOI:** 10.3389/fphar.2018.01226

**Published:** 2018-10-26

**Authors:** Jun Luo, Lei Huang, Aimei Wang, Yueyang Liu, Ruiping Cai, Weihong Li, Ming-Sheng Zhou

**Affiliations:** ^1^Department of Cardiology, The Affiliated Ganzhou Hospital of Nanchang University, Ganzhou, China; ^2^Department of Physiology, Shenyang Medical University, Shenyang, China; ^3^Department of Physiology, Jinzhou Medical University, Jinzhou, China

**Keywords:** cardiovascular diseases, endothelial nitric oxide synthesis, endoplasmic reticulum stress, resistin, vascular insulin signaling

## Abstract

**Background:** Endoplasmic reticulum (ER) stress plays an important role in the pathogenesis of obesity, insulin resistance and cardiovascular diseases (CVDs). Impairment of insulin vascular action may represent a mechanism linking insulin resistance and CVDs. The present study tested the hypothesis that adipocyte-derived resistin inhibits insulin-stimulated endothelial NO production through the induction of ER stress.

**Methods and Results:** Human umbilical vein endothelial cells (HUVC) were incubated with tunicamycin (an inducer of ER stress, 1–20 μg/mL) or resistin (10–100 ng/mL) for 1 h. Either tunicamycin or resistin increased GRP78 (an ER stress marker) expression associated with the impairment of insulin-stimulated Akt/eNOS phosphorylation, which were prevented by TUDCA (an ER stress suppressor). Resistin increased reactive oxygen species (ROS) production, antioxidant treatment inhibited resistin-induced GRP78 expression and impairment of insulin Akt/eNOS signaling, suggesting that ROS may involve resistin-induced ER stress. Resistin also increased JNK phosphorylation, which was prevented by TUDCA. JNK inhibitor SP600125 relieved the resistin inhibitory effects on endothelial insulin Akt/eNOS signaling. In *ex vivo* experiments, the incubation of aortic rings with resistin impaired insulin- but not acetylcholine-induced vasodilation, which was restored by TUDCA. LNAME (a NOS inhibitor) abolished insulin-induced vasorelaxation in the control or the resistin-treated aortic rings. In addition, resistin increased the mRNA expressions of proinflammatory cytokines tumor nuclear factor (TNF)α and interleukin (IL)-1β, which were also prevented by TUDCA.

**Conclusion:** Our results support the ideal that ER stress may play an important role for resistin impairment of vascular insulin signaling and insulin action. The mitigation of ER stress may represent a new strategy for prevention and treatment of CVDs in obesity and insulin resistant-related diseases.

## Introduction

The growing obesity epidemic and its associated metabolic dysfunction have made adipose tissue an important target of therapeutic interventions. Adipose tissue is not only an energy storage organ, but also an active endocrine organ that secretes many unique proteins known as adipokines such as resistin, adiponectin, leptin, tumor necrosis factor (TNF)α (Sidossis and Kajimura, 2015; Kumari et al., 2018). Under normal conditions, the adipokines released from adipose tissues may contribute to the maintenance of whole-body energy balance and glucose homeostasis (Sidossis and Kajimura, 2015). In obesity however, excessive expansion of adipose tissue may dysregulate the expressions (or secretions) of adipokines, leading to various metabolic abnormalities such as hyperlipidemia and hyperglycemia, which in turn induce insulin resistance and cardiovascular diseases (CVDs) (Lau et al., 2005; Bartelt and Heeren, 2014). It is well known that adipose tissue-derived hormones regulate insulin sensitivity in classic insulin-response tissues including the skeletal muscle and liver (Bartelt and Heeren, 2014). It has been reported that the majority of these adipocyte-derived hormones have important vascular effects (Verma et al., 2003; Gentile et al., 2008). Therefore, the changes in adipose tissue mass and metabolism may be a molecular link among insulin resistance, CVDs and visceral obesity (Verma et al., 2003; Fisman and Tenenbaum, 2014).

Resistin is an important adipokine that contributes to insulin resistance in mice. Resistin is exclusively expressed in the adipocytes of mice (Qatanani et al., 2009; Huang and Yang, 2016). Although human resistin is mainly produced by the macrophages rather than the adipocytes (Huang and Yang, 2016), experimental studies suggest that human resistin exacerbates adipose tissue inflammation and contributes to insulin resistance (Qatanani et al., 2009). In addition, resistin has important endothelial effects, including upregulating the expressions of endothelin 1, vascular cell adhesion molecule (VCAM1) and monocyte chemoattractant chemokine (MCP-1) and the impairment of vascular insulin-evoked vasorelaxation (Muse et al., 2004; Gentile et al., 2008).

Impaired vascular insulin signaling is proposed as an important contributor to the development of macro- and micro- vascular diseases in the patients with obesity or with type II diabetes (Schulman and Zhou, 2009; King et al., 2016). The impairment of insulin-evoked vasorelaxation by resistin may represent an important link between changes in adipokines and CVDs in obesity (Gentile et al., 2008). However, the mechanisms by which resistin impairment of vascular insulin action are still unclear. Insulin induces vasorelaxation via the stimulation of phosphoinositide (PI3K)/Akt/endothelial nitric oxide synthase (eNOS) pathway in endothelium (Schulman and Zhou, 2009; Zhou et al., 2009). Recent studies have shown that saturated fatty acids impair vasodilatory action of insulin via the activation toll-like receptor 4-induced endoplasmic reticulum (ER) stress (Kim et al., 2015). Emerging evidence suggests that ER stress is major link between insulin resistance and CVDs in obesity and type II diabetic mellitus (Kassan et al., 2012; Elmasry et al., 2018; Ghemrawi et al., 2018). It has been shown that ER stress in adipose tissue regulates the expressions of adipose tissue hormones adiponectin and resistin (Lefterova et al., 2009; Guo et al., 2017). ER stress impairs vascular insulin action (Zhou et al., 2012; Padilla and Jenkins, 2013). In the present study, we investigated whether resistin impaired vascular endothelial insulin PI3K/eNOS signaling and insulin vasodilator action via the induction of ER stress.

## Materials and Methods

### Materials

Human umbilical vein endothelial cell (HUVC) line was purchased from the Cell Bank of Shanghai (Shanghai, China). Human recombinant resistin, human recombinant insulin, acetylcholine chloride, tunicamycin, L-*N*^G^-nitro-L-arginine methyl ester (L-NAME), tauroursodeoxycholic acid (TUDCA), diphenyleneiodinium (DPI), N-acetylcysteine (NAC), nicotinamide adenine dinucleotide phosphate (NADPH), and lucigenin were purchased from Sigma-Aldrich (St. Louis, MO, United States). Rabbit polyclonal anti-peNOS (Ser 1177) and anti-pAkt (Ser473) antibodies were obtained from Cell Signaling Technology Inc. (Danvers, MA, United States), and anti-c-Jun N-terminal kinase (JNK), pJNK, anti-insulin receptor substance 1 (IRS1), anti-pIRS1(Ser 307), anti-glucose regulated protein 78 (GRP78), and anti-βactin antibodies from Santa Cruz Biotechnology Inco. (Santa Cruz, CA, United States). All other chemicals were of the best grade available from commercial sources.

### Vascular Reactivity Studies

Male C57BL/6 mice aged 8 weeks’ old were purchased from Beijing Charles River Animal Laboratory (Beijing, China). All animal studies complied with the international standards stated in the Guide for the Care and Use of Laboratory Animals. All animal experimental procedures were in accordance with the guidelines of the institution regarding animal research. After 2 weeks of accommodating to the new environment, the mice were anesthetized with ketamine (100 mg/kg) and xylazine (20 mg/kg) cocktail (I.P). The thoracic aorta was dissected and cleared of adhesive connective tissue. Aortic rings were mounted on the weir connected to isometric force transducers in the organ baths containing 5 mL physiological Krebs solution, and maintained at 37°C and bubbled with 95% oxygen. The rings were equilibrated under resting tension of 1 g for 90 min, then exposed to physiological Krebs solution containing 60 mmol/L KCl to induce vasoconstriction twice. Endothelium-dependent relaxation to acetylcholine (10^-9^–10^-5^mol/L) or vascular relaxation to insulin (10^-9^–10^-6^ mol/L) was determined in the aortic rings precontracted to 70% of maximal norepinephrine-induced vasoconstriction before and after 1 h of incubation with 100 ng/mL resistin or 100 μmol/L L-NAME (a NOS inhibitor). To test the role of ER stress in resistin impairment of insulin-induced vasodilation, the aortic rings were preincubated with TUDCA (500 μg/mL, a chemical chaperone) for 30 min before the incubation with resistin. Maximal response to an agonist (Emax) and the concentration of agonist required for a half-maximal response curve (ED_50_) were determined and calculated from the concentration-response curve, using best fit to a logistic sigmoid function.

### Cell Culture and Cell Suspension Protocols

HUVECs were seeded in 75 cm^2^ flasks and cultured in Dulbecco’s Modified Eagle’s Medium (DMEM) supplement with 10% fetal bovine serum (FBS) and 1% penicillin/streptomycin. The cells were maintained at 37°C, 95% humidity, and 5% CO_2_ and used between the passages 4 and 12. The cells were starved in DMEM medium with 1% FBS for 24 h before the cells were treated. To determine a dose response to resistin, the HUEVCs were incubated with different dose of resistin (10, 30, and 100 ng/mL) for 1 h. For cell suspension experiments, the cells were treated with 100 ng/mL resistin for 1 h followed by the incubation of 100 nmol/L insulin for 5 min in the presence or the absence of following compounds: (1) 500 μg/mL TUDCA; (2) NADPH oxidase inhibitor DPI (10 μmol/L); (3) antioxidant agent NAC (1 μmol/L); (4) JNK inhibitor SP600125 (12.5 μmol/L). All compounds were added into the medium 30 min before resistin treatment.

### Real-Time PCR

The cells were collected in 1 mL Trizol reagent. Total cellular RNA was isolated and quantitated. RNA (2 μg) was reverse-transcribed to cDNA using a superscript II RT first strand synthesis kit (Gibco, BRL) according to the manufacturer’s instructions. For the amplification, PCR was performed in total of 20 μL reaction mixture containing cDNA solution (80 ng), 0.1 μmol/L primer pair (sense primer and anti-sense prime) specific for TNFα (assay ID: Hs00174128-m1) or IL1β (Assay ID: Hs01336189-m1), and 0.2μmol/L fluorescence probe and PCR master mix assay kit (ABI) as previously described (Zhou et al., 2015). Relative quantities of each transcript were normalized by a housekeeping gene (GAPDH) and expressed as fold increase vs. control.

### Western Blot

The cells were harvested with lysis buffer containing a protein inhibitor cocktail. Protein content was quantified by Bio-Rad protein assay. Thirty microgram protein from each sample was loaded onto SDS-PAGE for the separation by electrophoresis, and proteins were transferred to nitrocellulose membranes. The membranes were incubated with blocking solution (5% milk added to TBST buffer) at room temperature for 1 h, and then incubated overnight at 4°C with specific primary rabbit polyclonal anti-peNOS (Ser 1177; 1:1000 dilution with blocking solution), anti-pAkt (Ser 473, 1:1000 dilution), anti-pIRS1 (Ser 307, 1:500 dilution), anti-IRS1 (1:500 dilution), anti-JNK (1:500 dilution), anti-pJNK (1:500 dilution) or anti-GRP78 (1:500 dilution) antibodies. After washing, the membranes were incubated with the appropriate secondary antibodies for 1 h at room temperature. The membrane was reblotted with anti- β-actin (1:500) antibody to serve as a loading control. Immune bands were detected by chemiluminescence and quantified by densitometry. Relative quantities of each protein were normalized by β-actin and expressed as fold increase vs. control.

### NADPH Oxidase Assay

HUEVCs were washed three times with Hanks’ balanced salt solution, and homogenized in 300 μL homogenization buffer (phosphate buffer 50 mmol/L, EDTA 0.01 mmol/L, leupeptin 2 μmol/L, pepstatin A 2 μmol/L, phenylmethylsulfonyl fluoride or phenylmethanesulfonyl fluoride 1 mmol/L, pH 7.4) by sonication. NADPH oxidase activity was determined by using lucigenin-enhanced chemiluminescence assay in the presence of NADPH (100 μmol/L) as substrate. In brief, 20 μL of cell homogenates were added into 50 mmol/L phosphate buffer (PH 7.4) containing 1 mmol/L EGTA as an assay solution, the reaction was triggered by adding NADPH (100 μmol/L) substrate and lucigenin (5 μmol/L). Protein content in homogenates was measured using the Bio-Rad method. The data was adjusted by protein and expressed as CPM/μg protein.

#### Statistical Analysis

The results were expressed as mean ± SEM. Statistical analyses were performed using SPSS 16.0 statistical software package (SPSS, Inc., Chicago, IL, United States), and statistical significance of difference was determined by one-way or two-way ANOVA with Bonferroni’s correction for multiple comparisons. Significance was considered present when p < 0.05.

## Results

### Tunicamycin Dose-Dependently Increased the Expression of GRP78 and Inhibited Insulin-Induced Phosphorylation of Akt and eNOS

**FIGURE 1:**
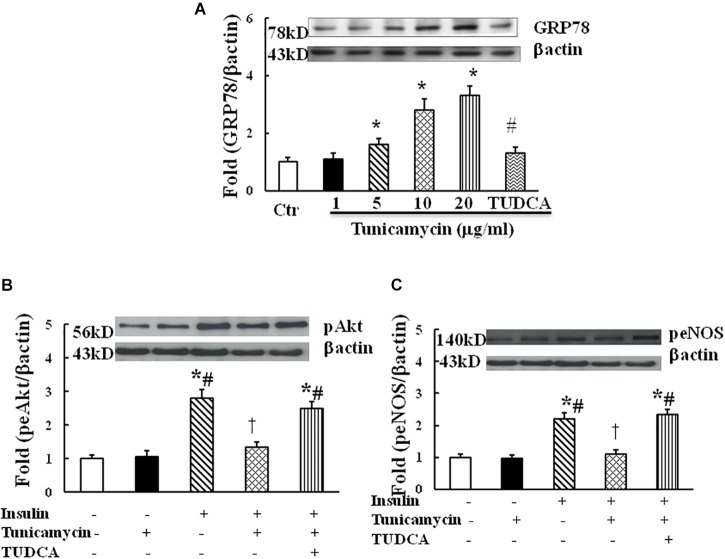
Effect of tunicamycin on the expression of GRP78 (**A**; *N* = 7 per condition), pAkt (Ser473, **B**; *N* = 7 per condition), peNOS (Ser1177, **C**; *N* = 7 per condition) in HUVECs. Tunicamycin (1–20 μg/mL) dose-dependently increased GRP78 expression, which was prevented by TUDCA (500 μg/mL) treatment. Tunicamycin (20 μg/mL) inhibited insulin-stimulated Akt and eNOS phosphorylation, TUDCA restored the expressions of pAkt and peNOS induced by tunicamycin. Data was presented as mean **±** SE. Ctr, control; GRP78, glucose regulated protein 78. Loading control imagines for βactin were reused for pAkt and peNOS. ^∗^*p* < 0.05, vs. control group, ^#^*p* < 0.05, vs. correspondence tunicamycin group, ^†^*p* < 0.05 vs. insulin group.

Tunicamycin is an ER stress inducer. To examine whether ER stress impairs insulin signaling in endothelium, HUVECs were incubated with vehicle (0.02% DMSO) and tunicamycin at the dose of 1, 5, 10, and 20 μg/mL dissolved in 0.02% DMSO solution for 1 h, tunicamycin dose-dependently increased the protein expression of GRP78, an ER stress marker (Figure 1A). Next, HUVECs were incubated with the vehicle, tunicamycin (20 μg/mL) with or without TUDCA (500 μg/mL) for 1 h followed the incubation with insulin (100 nmol/L) for 5 min. Insulin stimulated Akt (ser473) and eNOS (Ser1177) phosphorylation, tunicamycin significantly inhibited insulin-induced Akt (Ser 473) and eNOS (Ser 1171) phosphorylation, which were restored by TUDCA treatment (Figures 1B,C). These results suggest that tunicamycin-induced ER stress can inhibit insulin-stimulated Akt and eNOS pathway in endothelium.

### Resistin-Induced ER Stress Contributed to the Impairment of Endothelial Insulin Signaling

**FIGURE 2:**
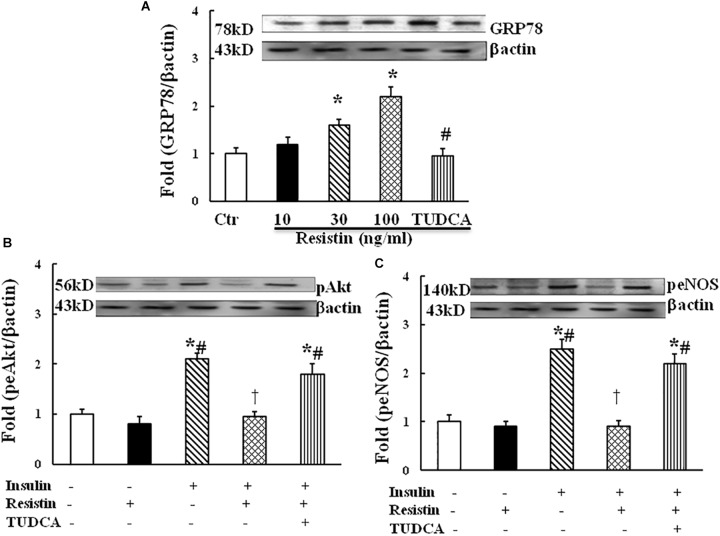
Effect of resistin on the expression of GRP78 (**A**, *N* = 7, per condition), pAkt (Ser473, **B**; *N* = 7, per condition), peNOS (Ser1177, **C**; *N* = 7, per condition) in HUVECs. Resistin (10–100 ng/mL) increased GRP78 expression in a dose-dependent manner, which was reduced by TUDCA treatment (500 μg/mL). Resistin (100 ng/mL) inhibited insulin-stimulated pAkt (Ser473) and peNOS (Ser1177) expression, TUDCA prevented an increase in the expression of pAkt and peNOS induced by resistin. Loading control imagines for βactin were reused for pAkt and peNOS. ^∗^*p* < 0.05, vs. control group, ^#^*p* < 0.05, vs. correspondence resistin group, ^†^*p* < 0.05 vs. insulin group.

To determine the effect of resistin on ER stress, HUVECs were exposed to different doses of resistin (10, 30, and 100 ng/mL) for 1 h. Resistin increased the protein expression of ER stress marker GRP87 in a dose-dependent manner (Figure 2A). Resistin inhibited insulin-induced Akt (Ser473) and eNOS (Ser1177) phosphorylation, ER stress suppressor TUDCA (500 μg/mL) reversed the inhibitory effects of resistin on insulin signaling through Akt and eNOS phosphorylation (Figures 2B,C), suggesting that resistin inhibits endothelial insulin Akt/eNOS signaling via the induction of ER stress.

### Interaction Among Reactive Oxygen Species (ROS), ER Stress, and Insulin Signaling in the Resistin-Treated HUVECs

**FIGURE 3:**
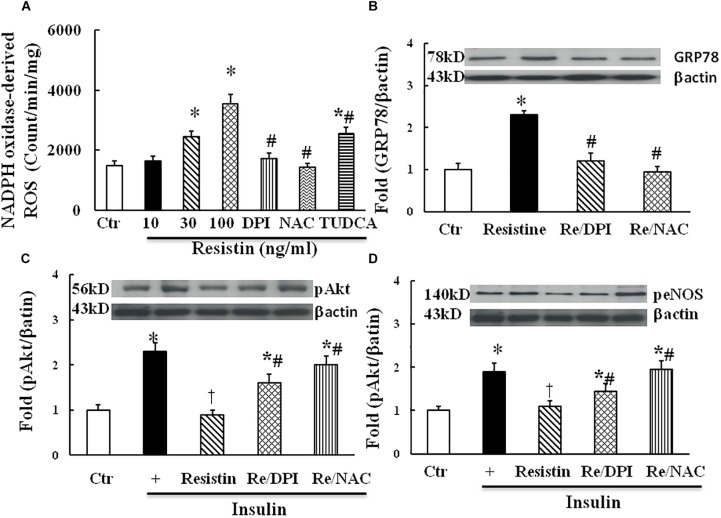
Effect of antioxidant on resistin-induced ROS (**A**, *N* = 7 per condition), GRP78 (**B**, *N* = 7 per condition), pAkt (Ser473, **C**; *N* = 7, per condition) and peNOS (Ser1177, **D**; *N* = 7, per condition) expressions in HUVECs. Resistin (10–100 ng/mL) dose-dependently increased NADPH oxidase-derived ROS production, which was partially reduced by TUDCA (500 μg/mL). Antioxidant with either NADPH oxidase inhibitor DPI (10 μmol/L) or ROS scavenger NAC (1 μmol/L) inhibited resistin-induced GRP78 expression and improved insulin signaling pAkt (Ser473) and peNOS (Ser 1177) impaired by resistin. Loading control imagines for βactin were reused for pAkt and peNOS. ^∗^*p* < 0.05, vs. control, ^#^*p* < 0.05, vs. correspondence resistin group, ^†^*p* < 0.05 vs. insulin group.

Both ROS and ER stress are important components of intracellular stress, overproduction of ROS within ER is a major cause of ER stress (Ghemrawi et al., 2018). As shown in Figure 3A, resistin dose-dependently increased NADPH oxidase-derived ROS production. Antioxidants with either DPI (10 μmol/L) or NAC (1 μmol/L) prevented an increase in NADPH oxidase-derived ROS, and TUDCA (500 μg/mL) partially reduced NADPH-derived ROS production in the resistin-treated cells. Moreover, antioxidants with either DPI or NAC prevented the increase in resistin-induced GRP78 expression (Figure 3B) and improved insulin-stimulated Akt and eNOS phosphorylation in the resistin-treated cells (Figures 3C,D). These results suggest that resistin induces cell stresses ROS and ER stress, which interact to impair endothelial insulin signaling.

### Resistin Impaired Insulin Signaling Through ER Stress-Mediated JNK Activation

**FIGURE 4:**
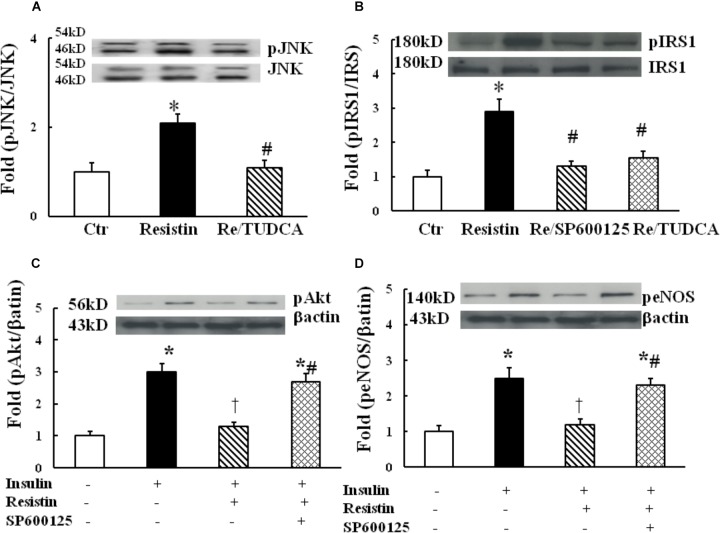
Effect of JNK inhibition on insulin signaling through IRS1/Akt/eNOS pathway in the resistin-treated HUEVCs. **(A)** Resistin (100 ng/mL) increased JNK phosphorylation, which was prevented by TUDCA (*N* = 7, per condition); **(B)** TUDCA (500 μg/mL) or JNK inhibitor (12.5 μmol/L) prevented resistin-induced IRS phosphorylation at serine residue (Ser 307); **(C,D)** JNK inhibitor Sp600125 (12.5 μmol/L) improved insulin signaling molecules pAkt (Ser 473), peNOS (Ser1177) impaired by resistin (*N* = 7, per condition). ^∗^*p* < 0.05, vs. control, ^#^*p* < 0.05, vs. correspondence resistin group, ^†^*p* < 0.05 vs. insulin group.

It has been shown that the activation of JNK pathway can inhibit insulin PI3K/Akt signaling, and the underlying mechanisms may involve IRS1 phosphorylation at serine residues (Aguirre et al., 2000). To determine the role of JNK in resistin inhibition of endothelial insulin signaling, HUVECs were exposed to 100 ng/mL resistin for 1 h. As shown in Figure 4A, resistin significantly increased JNK phosphorylation, which was prevented by TUDCA, suggesting that resistin activates JNK via ER stress. Resistin increased pIRS (Ser307), which was prevented by either JNK inhibitor Sp600125 (12.5 μmol/L) or TUDCA (Figure 4B). Furthermore, Sp600125 prevented the decrease in the resistin-induced pAkt (Ser 473) and peNOS (Ser 1177) (Figures 4C,D).

### TUDCA Prevented the Increase in the Expressions of Resistin-Induced Proinflammatory Genes TNFα and IL1β

**FIGURE 5:**
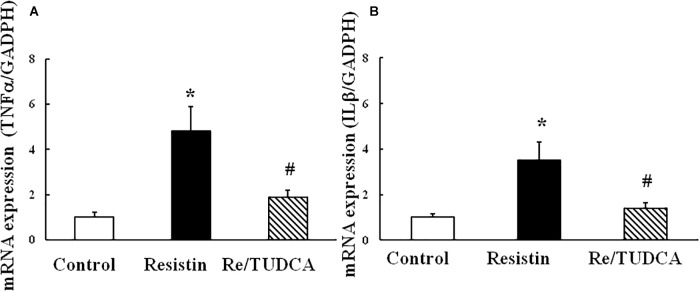
Effect of resistin on the mRNA expression of proinflammatory genes TNFα (**A**, (*N* = 7, per condition) and IL1β (**B**, *N* = 7 per condition) in HUVECs. ^∗^*p* < 0.05, vs. control group, ^#^*p* < 0.05, vs. resistin group.

As shown in Figure 5, resistin (100 ng/mL) increased the mRNA expression of proinflammatory genes TNFα and IL1β, and TUDCA reversed the resistin-induced changes in the mRNA expressions of TNFα and IL1β.

### Resistin-Induced ER Stress Contributed to Impaired Insulin-Evoked Vascular Relaxation

**FIGURE 6:**
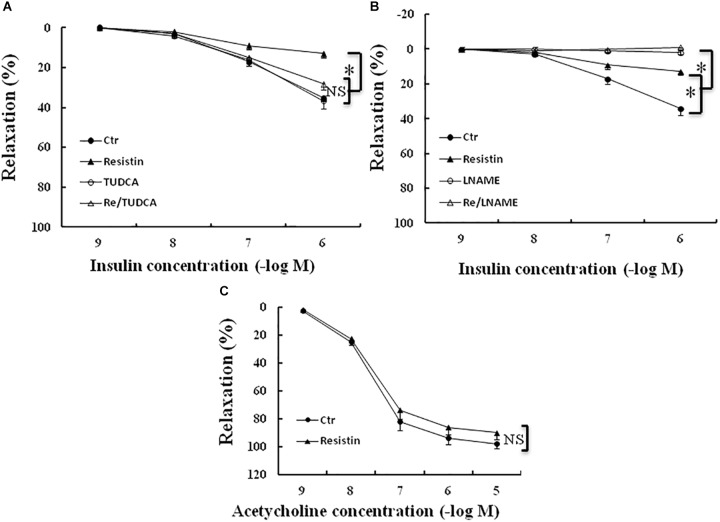
Effect of resistin on acetylcholine- or insulin-induced vasodilation in the aortic rings. The incubation with resistin (100 ng/mL) ex vivo significantly attenuated insulin-induced vasorelaxation in the aortic rings **(A,B)**, but did not impair acetylcholine-induced vasorelaxation (**C**, *N* = 8 aortic ring per group). TUDCA (500 μg/mL) significantly improved vascular relaxant response to insulin in resistin-treated aortic rings (**A**, *N* = 8 aortic rings per group), LNAME (100 μmol/L) abolished insulin-induced vasorelaxation in either vehicle or resistin-treated aortic rings (**B**, *N* = 8 aortic rings per group). ^∗^*p* < 0.05, NS: no significant difference.

It has been shown that resistin inhibits insulin-induced vasodilation (Gentile et al., 2008). To determine the role of ER stress in the resistin impairment of insulin-evoked vasodilation, the aortic rings were preincubated with resistin (100 ng/mL) for 1 h in the presence or the absence of TUDCA (500 μg/mL). The aortic rings were preconstracted with norepinephrin (about 30 nmol/L). There were no significant differences in norepinephrin-induced contraction force among the control (5.2 **±** 0.4 mN), TUDCA (5.8 **±** 0.5 mN), resistin (5.5 **±** 0.6 mN) and resistin/TUDCA (5.6 **±** 0.4 mN) groups (all *p* > 0.05). Resistin significantly attenuated insulin-induced vasorelaxation, as demonstrated by Emax (14 **±** 2% vs. 35 **±** 3% in the control group, *p* < 0.05), but there was no significant difference in ED50 between resistin and control group (7.3 **±** 0.2 vs. 7.0 **±** 0.1 (-log molar) in the control group, *p* > 0.05). TUDCA significantly improved insulin-induced vasorelaxation in the resistin-pretreated aortic rings (Emax: 14 **±** 3% vs. 29 **±** 3% in the control group, *p* < 0.05; ED50: 7.1 **±** 0.1 vs. 7.3 **±** 0.2 (-log molar), *p* > 0.05; Figure 6A), L-NAME abolished insulin-induced vasodilation in the vehicle and the resistin-treated aortic rings (Figure 6B). These results suggest that resist impairs insulin-evoked vasodilation via the induction of ER stress. Furthermore, we investigated the effects of resistin on acetylcholin-induced vasorelaxation *ex vivo*. Resistin did not significantly impair acetylcholine-induced vasorelaxation (Emax: 92 **±** 4% vs. 98 **±** 2% in the control group; ED50: 7.6 **±** 0.2 vs. 7.7 **±** 0.3 (-log molar) in control group, all *p* > 0.05; Figure 6C).

## Discussion

Both adiopkine resistin and ER stress are proposed to be associated with insulin resistance and CVDs in obesity and type II diabetic mellitus (Schwartz and Lazar, 2011; Ghemrawi et al., 2018). Loss of endothelial insulin-induced vasorelaxation is considered an important linking insulin resistance and CVDs in obesity and metabolic diseases (Schulman and Zhou, 2009). It has been shown that resistin impairs insulin-evoked vasorelaxation (Gentile et al., 2008). However, the underlying mechanims by which resistin impairs endothelial insulin signaling and vascular action are still not elucidated. There are also no reports in literature that resistin induces ER stress in the endothelium or other vascular cells. In the present study, we, for the first time, demonstrated that resistin induced ER stress in endothelial cells, which subsequently resulted in the impairment of endothelial insulin Akt/eNOS pathway and insulin vasodilator action. Furthermore, our results showed that resistin-induced ER stress also contributed to increased the expressions of proinflammatory genes TNFα and IL1β1 in endothelial cells. These results reveal a novel mechanism that resistin impairs vascular insulin action and endothelial insulin signaling via the induction of ER stress.

Obesity is associated with increased CV risk. Excess expansion of adipocytes alters the production of various adipocyte-derived factors which may contribute to insulin resistance and CV diseases in obesity (Park et al., 2017; Kumari et al., 2018). Resistin is an important adipocyte-derived hormone, which has been shown to induce insulin resistance in obesity (Codoner-Franch et al., 2014; Asgary et al., 2015). With the exception of the inhibition of metabolic insulin sensitivity, resistin also impairs insulin-mediated vasodilator effects (Verma et al., 2003; Dick et al., 2006; Park et al., 2017). Insulin induces vasodilator effects via the activation of endothelial PI3K/Akt/eNOS pathway (Steinberg et al., 1994). Constant stimulation of endothelium NO by insulin may be critical for the maintenance of vascular health as well as glucose homeostasis, because insulin-induced NO is an important cardiovascular protective molecule and insulin-induced NO production is critical for the regulation of blood flow and glucose dispose in the skeletal muscle (Schulman and Zhou, 2009). Therefore, resistin impairment of insulin Akt/eNOS pathway may be an important convergence point linking CV and metabolic diseases (Codoner-Franch et al., 2014; Park et al., 2017). The present study provided convincing evidences that resistin impaired endothelial insulin signaling through the induction of ER stress as TUDCA, a chemical chaperone, significantly prevented the increase in resistin-induced ER stress and reversed resistin inhibitory effects on insulin Akt/eNOS pathway and insulin-mediated vasodilation.

It has been suggested that ER stress in adipocytes may inhibit insulin signaling through the activation of inositol- requiring enzyme 1α (IRE1α) (Khan and Wang, 2014). IRE1α constitutes one important player of the unfloding protein response (UPR) under chronic ER stress. IRE1α promotes JNK phosphorylation (Ghemrawi et al., 2018), which impairs insulin PI3K/Akt signaling via increasing the serine phosphorylation and decreasing the tyrosine phosphorylation of IRS-1 (Aguirre et al., 2000). The present study supports the ideal that resistin-induced ER stress inhibits endothelial insulin Akt/eNOS signaling via the activation of JNK, which increased ISR1 phosphorylation at serine residue.

Emerging evidence suggests that chronic cellular stress such as ER stress and oxidative stress play important roles in the pathogensis of insulin resistance and CVDs in obesity and type II diabetes (Civelek et al., 2009; Elmasry et al., 2018; Ghemrawi et al., 2018). There is a complex interaction between ROS and ER stress (Ghemrawi et al., 2018; Kritsiligkou et al., 2018; Mukaigasa et al., 2018), and ER is the place for Nox NADPH oxidase synthesis and activation (Laurindo et al., 2014). ROS is produced during protein folding process within ER (Ghemrawi et al., 2018), and is considered as an integral component of unfloding protein response (UPR) signaling. Excess ROS can in turn trigger the UPR and induce ER stress (Malhotra and Kaufman, 2007; Cao and Kaufman, 2014; Luchetti et al., 2017). It has been shown that resistin increase ROS production which causes vascular cell dysfunction (Raghuraman et al., 2016). Here we showed that resistin increased ROS which caused ER stress and UPR in endothelium. ER stress in turn further promoted ROS production. These results suggest that resistin may increase ROS generation and oxidative stress, and create a self-perpetuating vicious cycle, which concomitantly contribute to the impairment of endothelial insulin Akt/eNOS signaling.

Obesity and CVDs are always associated with chronic low-grade inflammation in the adipose tissues or the vasculatures. The interplay between ER and oxidative stress may promote inflammation due to the activation of UPR cascade (Zhang et al., 2017; Ghemrawi et al., 2018). As mentioned above, IRE1α, an important player of UPR, promotes the activation of nuclear factor (NF)κB (Ghemrawi et al., 2018). NFκB is a primary regulator of inflammatory responses, increasing transcriptional activity of at least 125 proinflammatory genes including TNFα and IL1β (Tak and Firestein, 2001). Here we showed that that ER stress induced by resistin also increased the expressions of proinflammatory genes TNFα and IL1β, suggesting that ER stress may involve resistin promotion of endothelial activation and inflammation.

### Limitation

Human and mouse resistin differ in the major site of their production, gene and protein sequences; thus, there may exist interspecies difference for their biological effects (Qatanani et al., 2009; Schwartz and Lazar, 2011; Huang and Yang, 2016). In the present study, we investigated human resistin biological effects in two species: insulin signaling effect in human endothelial cells (HUVCs) and insulin vasorelaxant action in the mouse aorta. It should be a limitation for our study, although several studies have shown that there are conserved biological effects of resistin across the species such as insulin resistance (Qatanani et al., 2009).

In summary, the present study provides evidence that adipokine resistin induces ER stress and ROS, which contributes to the impairment of insulin-stimulated Akt/eNOS signaling in endothelium and the attenuation of insulin-induced vasodilator effects. Resistin-induced ER stress may also promote endothelial inflammation and activation. ER stress induced by resistin may play an important role in the pathogenesis of vascular dysfunction or vascular diseases associated with obesity or other metabolic disorders. The mitigation of ER stress may be beneficial effects on CV system in obesity and metabolic diseases.

## Author Contributions

JL contributed to the conception and design of the work, acquisition of data, analysis and interpretation of data, statistical analysis. LH and AW contributed to acquisition of data, analysis and interpretation of data, statistical analysis. YL, RC, and WL contributed to acquisition of data, analysis and interpretation of data. M-SZ contributed to the conception and design of the work, analysis and interpretation of data and draft of the manuscript.

## Conflict of Interest Statement

The authors declare that the research was conducted in the absence of any commercial or financial relationships that could be construed as a potential conflict of interest.
