# Corrigendum: Resistin-Induced Endoplasmic Reticulum Stress Contributes to the Impairment of Insulin Signaling in Endothelium

**DOI:** 10.3389/fphar.2018.01446

**Published:** 2018-12-10

**Authors:** Jun Luo, Lei Huang, Aimei Wang, Yueyang Liu, Ruiping Cai, Weihong Li, Ming-Sheng Zhou

**Affiliations:** ^1^Department of Cardiology, The Affiliated Ganzhou Hospital of Nanchang University, Ganzhou, China; ^2^Department of Physiology, Shenyang Medical University, Shenyang, China; ^3^Department of Physiology, Jinzhou Medical University, Jinzhou, China

**Keywords:** cardiovascular diseases, endothelial nitric oxide synthesis, endoplasmic reticulum stress, resistin, vascular insulin signaling

An author name was incorrectly spelled as Jun Lou. The correct spelling is Jun Luo. The authors apologize for this error and state that this does not change the scientific conclusions of the article in any way. The original article has been updated.

In the published article, there was an error in affiliation 1. Instead of, the Department of Cardiology, Affiliated Ganzhou City Hospital, Nanchang University, it should be, the Department of Cardiology, The Affiliated Ganzhou Hospital of Nanchang University.

In addition, there was an error in the affiliation(s) for Ming-Sheng Zhou. Instead of, 2 Department of Physiology, Shenyang Medical University, Shenyang, China, 3 Department of Physiology, Jinzhou Medical University, Jinzhou, China, they should only have, 2 Department of Physiology, Shenyang Medical University, Shenyang, China. The authors apologize for this error and state that this does not change the scientific conclusions of the article in any way. The original article has been updated.

## Conflict of Interest Statement

The authors declare that the research was conducted in the absence of any commercial or financial relationships that could be construed as a potential conflict of interest.

